# Editorial: “Obesity, Metabolism and the immune system”

**DOI:** 10.3389/fragi.2022.1016274

**Published:** 2022-09-08

**Authors:** Daniela Frasca, Leena P. Bharath, Barbara Nikolajczyk

**Affiliations:** ^1^ University of Miami Miller School of Medicine, Miami, FL, United States; ^2^ Merrimack College, North Andover, MA, United States; ^3^ University of Kentucky, Lexington, KY, United States

**Keywords:** obesity, metabolism, aging, inflammation, immunity

The focus of the field of immunometabolism is to understand the metabolic pathways that play crucial roles in immune cell function. Although the study of immune cell metabolism dates back decades, metabolism and immunity have been traditionally considered two separate systems with distinct functions: metabolism regulating the transformation and assimilation of nutrients, and the immune system regulating the capacity of an individual to respond to infections. However, ongoing research reveals that these two systems work together to regulate the individual’s response to stress, and immunometabolic studies represent a rapidly emerging and evolving field of research. Our aim with this Research Topic is to highlight the current understanding of the role played by immune cell metabolism in regulating cell/tissue function aging and the associated comorbidity of obesity/diabetes.

It is well known that effector cell function is intrinsically linked to cell metabolism and it has been shown that metabolic enzymes and their regulators can also have a direct effect in the regulation of broad array of cellular functions. Cell activation is critically supported by metabolic shifts that fuel multiple aspects of cell activation and differentiation, suggesting the exciting possibility that cell function under various conditions, including aging and obesity, could be regulated by manipulating the cell’s metabolism.

Numerous changes occur in the human immune system with advancing age, many of which are first detectable around the sixth decade of life. Aging is often associated with a progressive decline in health resulting in the loss of the quality of life that limits health-span, defined as number of years spent in good health. One critical goal of aging research is to increase health-span. The understanding of dysregulated immune cell metabolism as an elemental cause of many age-related conditions and diseases is gaining attention in recent years. How metabolic alterations accumulate during the aging process in the different immune cells, and how those changes may impact mechanisms underlying age-related metabolic decline is not yet known.

This Research Topic includes 4 contributions (one mini-review, one review and two research papers) from experts in the field. Here is a summary of the single contributions.

The mini-review “Aging effects on epicardial adipose tissue” by G. Iacobellis summarizes the characteristics of the epicardial adipose tissue (EAT), and shows that aging changes both the function and the morphology of the EAT and induces apoptotic and fibrotic changes that alter its transciptomic and proteomic profile. It is still not clear, however, if these changes are due to aging alone or together with age-related chronic diseases such as the coronary artery disease.

The review “Implications of inflammatory states on dysfunctional immune responses in aging and obesity” by A. Thomas and collaborators summarizes shared and distinct features of aging and obesity, two conditions characterized by chronic, low-grade systemic inflammation and dysfunctional immunity and associated to several clinical problems, including reduced vaccine responses. The cited work favors the hypothesis that obesity is a state of accelerated immune aging.

The original research paper “Altered nutrient uptake causes mitochondrial dysfunction in senescent CD8^+^ EMRA T cells during type-2 diabetes” by L. Callender and collaborators shows features of mitochondria in CD8^+^ EMRA T cells from patients with type-2 diabetes, as compared to those from healthy controls. These mitochondria are highly metabolic, characterized by higher oxidative capacity and ROS, as well as higher lipid droplet accumulation, due to lower fatty acid oxidation. These senescent-like mitochondria are associated with dysfunctional immune responses, thus highlighting similarities between immune cell characteristics in aging and obesity.

The original research paper “Obesity and fatty acids promote mitochondrial translocation of Stat3 through ROS-dependent mechanisms” from R. Conway and collaborators shows that obesity induces persistent activation and translocation of Stat3 in mitochondria (mitoStat3) of CD4^+^ T cells, leading to altered cellular redox homeostasis, nutrient metabolism and immune responses. MitoStat3 activation depends on fatty acids, and its pharmacological inhibition by a mitochondria-targeted Stat3 inhibitor or by a ROS scavenger decreases fatty-acid-induced secretion of the pro-inflammatory cytokines IL-17A and IL-6, demonstrating a link between mitoStat3 and pro-inflammatory cytokine secretion. This mechanism shares similarities with changes in STAT3 activation in CD4^+^ T cells from older subjects per previous work from this group.

Taken together, this series extends the current understanding of immunometabolism in aging and obesity, while identifying new mechanisms foundational to the interrelationship between these conditions. The editors are optimistic that the assembled series will spark more research into actionable changes in immunometabolism that support the clinical problems triggered by these naturally occurring events.

